# Inter-Regulation of K_v_4.3 and Voltage-Gated Sodium Channels Underlies Predisposition to Cardiac and Neuronal Channelopathies

**DOI:** 10.3390/ijms21145057

**Published:** 2020-07-17

**Authors:** Jérôme Clatot, Nathalie Neyroud, Robert Cox, Charlotte Souil, Jing Huang, Pascale Guicheney, Charles Antzelevitch

**Affiliations:** 1 Department of Cardiovascular Research, Lankenau Institute for Medical Research, Wynnewood, PA 19096, USA; CoxR@mlhs.org (R.C.); AntzelevitchC@mlhs.org (C.A.); 2 Division of Neurology, The Children’s Hospital of Philadelphia, Abramson Research Center, Room 512C-D, 3615 Civic Center Boulevard, Philadelphia, PA 19104, USA; 3 Team “Genomics and Pathophysiology of Myocardial Diseases”, Faculté de Médecine Pitié-Salpêtrière, 91 Boulevard de l’Hôpital, Sorbonne Université, UMR_S1166, F-75013 Paris, France; nathalie.neyroud@sorbonne-universite.fr (N.N.); charlotte.souil@gmail.com (C.S.); pascale.guicheney@sorbonne-universite.fr (P.G.); 4 Team “Genomics and Pathophysiology of Myocardial Diseases”, Faculté de Médecine Pitié-Salpêtrière, 91 Boulevard de l’Hôpital, INSERM, UMR_S1166, F-75013 Paris, France; 5 Institute of Cardiometabolism and Nutrition, ICAN, Pitié-Salpêtrière Hospital, 47-83 Boulevard de l’Hôpital, F-75013 Paris, France; 6 Department of Biostatistics, Epidemiology and Informatics, University of Pennsylvania Perelman School of Medicine, Philadelphia, PA 19104, USA; jing14@pennmedicine.upenn.edu; 7 Lankenau Heart Institute, Main Line Health System, Wynnewood, PA 19096, USA; 8 Sidney Kimmel Medical College, Thomas Jefferson University, Philadelphia, PA 19104, USA

**Keywords:** arrhythmia, Brugada syndrome, spinocerebellar ataxia, Na_v_1.5, *SCN5A*, Kv4.3, *KCND3*, *SCN1A*, Na_v_1.1, channelopathies

## Abstract

Background: Genetic variants in voltage-gated sodium channels (Na_v_) encoded by *SCNXA* genes, responsible for I_Na_, and K_v_4.3 channels encoded by *KCND3*, responsible for the transient outward current (I_to_), contribute to the manifestation of both Brugada syndrome (BrS) and spinocerebellar ataxia (SCA19/22). We examined the hypothesis that K_v_4.3 and Na_v_ variants regulate each other’s function, thus modulating I_Na_/I_to_ balance in cardiomyocytes and I_Na_/I_(A)_ balance in neurons. Methods: Bicistronic and other constructs were used to express WT or variant Na_v_1.5 and K_v_4.3 channels in HEK293 cells. I_Na_ and I_to_ were recorded. Results: *SCN5A* variants associated with BrS reduced I_Na_, but increased I_to_. Moreover, BrS and SCA19/22 *KCND3* variants associated with a gain of function of I_to_, significantly reduced I_Na_, whereas the SCA19/22 *KCND3* variants associated with a loss of function (LOF) of I_to_ significantly increased I_Na_. Auxiliary subunits Na_v_β1, MiRP3 and KChIP2 also modulated I_Na_/I_to_ balance. Co-immunoprecipitation and Duolink studies suggested that the two channels interact within the intracellular compartments and biotinylation showed that LOF *SCN5A* variants can increase K_v_4.3 cell-surface expression. Conclusion: Na_v_ and K_v_4.3 channels modulate each other’s function via trafficking and gating mechanisms, which have important implications for improved understanding of these allelic cardiac and neuronal syndromes.

## 1. Introduction

Variants in *SCN5A*, the gene encoding the cardiac voltage-gated sodium channel, Na_v_1.5, have been associated with life-threatening arrhythmia syndromes, including Brugada syndrome (BrS). BrS is an inherited cardiac channelopathy associated with a high risk of ventricular tachycardia and fibrillation leading to sudden cardiac death. The typical BrS electrocardiographic (ECG) pattern is characterized by the presence of prominent J waves appearing as ST-segment elevation, usually limited to the right precordial ECG leads, V1-V3 [1]. This ECG phenotype has been attributed to LOF variants in inward currents such as I_Na_ or to gain of function (GOF) variants in outward repolarizing currents such as I_to_ [2,3]. Interestingly, Portero et al. recently reported that expression of K_v_4.3 can reduce I_Na_ [4]. A fine balance may thus exist between these two currents during the early phases of the action potential (AP). An increase in I_to_ associated with a GOF variants in K_v_4.3, can simultaneously lead to LOF of I_Na_. The reduced levels of I_Na_ affects the upstroke (phase 0) and in combination with augmented levels of I_to_ can accentuate phase 1 of the action potential and the phenotypic expression of BrS. K_v_4.3 is also highly expressed in the brain and contributes to A-type current (I_A_) involved in the repolarization phase of the action potential of neurons. I_A_ regulates subthreshold dendritic excitability and modulates dendritic calcium influx via voltage gated calcium channels in Purkinje cells. K_v_4.3 LOF variants lead to repolarization defects and reduced cellular excitability, giving rise to spinocerebellar ataxia SCA19/22. Whereas LOF variants in *KCND3* have been linked to SCA19/22, GOF variants have been associated with BrS. Interestingly, some *KCND3* GOF variants (e.g., L450F) have been associated with both BrS and spinocerebellar ataxia SCA19/22 [5]. The reason for this dichotomy is not known and the ionic and cellular basis for SCA19/22 is not well defined. The present study examines the hypothesis that voltage-gated sodium (I_Na_) and K_v_4.3 (I_to_) channels modulate each other’s function and that this inter-regulation is mediated by interaction of both α and β subunits forming a megacomplex or channelosome. To do so, we have selected well-characterized genetic variants that have been implicated in Brugada and/or spinocerebellar ataxia SCA19/22 syndromes. The non-conducting mutants R878C, G1743R and E555X of Na_v_1.5 respectively gating-deficient, trafficking-deficient and missense variant leading to a premature stop codon identified in BrS patients were selected. The GOF Kv4.3-L450F identified in BrS and SCA19/22 and the LOF K_v_4.3-Δ227F associated with SCA19/22 were selected for study as well. In HEK293 cell line, we examined the effects of genetic variants in *SCN5A* associated with BrS on K_v_4.3 function by examining the effect of Na_v_1.5 trafficking-deficient to Na_v_1.5 trafficking-efficient channels on I_to_ [2,3,6,7]. We then examined the effects of *KCND3* variants associated with BrS and spinocerebellar ataxia SCA19/22, on both Na_v_1.5 and Na_v_1.1 function by examining the effect of K_v_4.3 trafficking-deficient vs trafficking-efficient channels on I_Na_. Finally, we examined regulation of the I_Na_/I_to_ balance secondary to expression of the different auxiliary subunits, including: Na_v_beta1, MiRP3 and KCNIP2.

## 2. Results

### 2.1. R878C, G1743R and E555X Na_v_1.5 Variants Affect I_to_

**Figure 1:**
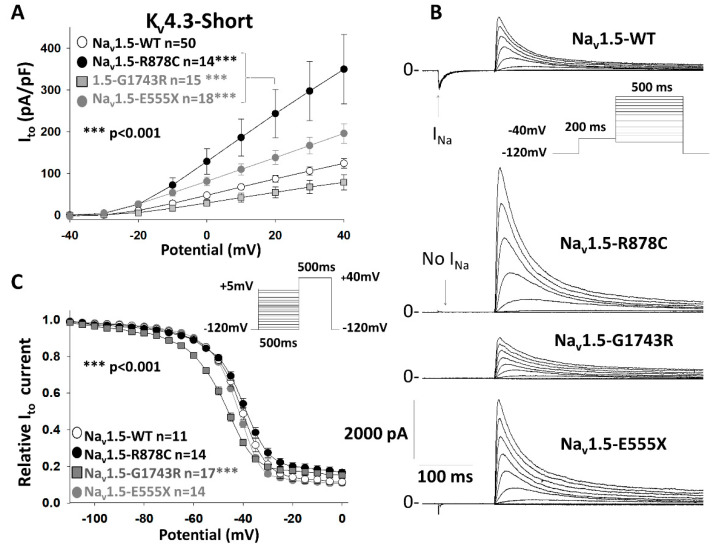
The presence of Na_v_1.5 variants affects outward current (I_to_). (**A**) I_to_ current–voltage relationships recorded from HEK293 cells co-expressing K_v_4.3-short (pGFP-IRES-KCND3-Short) channels and either WT, R878C, G1743R, or E555X-Na_v_1.5 channels (pcDNA3.1-GFP-SCN5A). (**B**): Representative current traces of I_Na_ in the prepulse followed by I_to_. Inset shows the voltage protocol employed. The presence of Na_v_1.5 variants significantly affect I_to_ compared to WT, at + 20 mV, *** *p* < 0.001 for the three variants (In pA/pF; WT = 87.14 ± 8.2, R878C = 243.3 ± 57.55, G1743R = 54.8 ± 12.9, E555X = 138 ± 16.8) (**C**): I_to_ steady-state inactivation. Note: G1743R-Na_v_1.5 significantly shifts the steady-state inactivation V_1/2_ of I_to_ compared to WT-Na_v_1.5, *** *p* < 0.001. *n* represents the number of recorded cells. In panel A, *n* = 50 WT cells correspond to total of WT cells patched against each variant.

I_Na_ and I_to_ were recorded from HEK293 cells 36 h after co-transfection with pGFP-*KCND3* and pGFP-*SCN5A*-WT, R878C or G1743R. None of the cells expressing the Na_v_1.5 variants displayed I_Na_, (Supplementary Figure S1) consistent with previous reports by us and others showing that R878C and G1743R variants in *SCN5A* abolish I_Na_ [2,8,9,10]. It is noteworthy that we previously established that E555X mutation leads to expression of non-functioning truncated channels comprised of only the first domain [11]. Interestingly, in the cells expressing variant Na_v_1.5 channels, peak I_to_ was significantly increased when compared to cells expressing the WT Na_v_1.5 channel (Figure 1A,B and Table 1). The Na_v_1.5-R878C gating-deficient but trafficking efficient channel, in addition to abolishing I_Na_ due to major pore dysfunction was associated with the largest increase in I_to_. These effects are consistent with the conditions known to give rise to the BrS phenotype. Interestingly, the Na_v_1.5-G1743R trafficking-deficient channel led to a significant 62.9% decrease of peak I_to_, compared to Na_v_1.5-WT due to a −6.2 mV shift of steady-state inactivation (Figure 1A,C and Table 1). Indeed, the −40 mV prepulse used for the I-V curves in Figure 1B, represented by the vertical bar in Figure 1C, led to a greater inactivated fraction of K_v_4.3 channels, 67.3% of WT explaining the decrease in I_to_ observed on the I-V curve (Figure 1B and Table 1). I_to_ recovery from inactivation was recorded but no significant difference was noted between WT and any of the *SCN5A* variant channels (Supplementary Figure S2).

In order to ascertain whether this effect could be due to the short isoform of K_V_4.3, another set of experiments was performed using pcDNA3.1-GFP-Na_v_1.5 and pGFP-K_v_4.3-long isoform (Supplementary Figure S3). The effects observed on peak I_to_ were identical suggesting that these effects are independent of the isoform used.

### 2.2. LOF and GOF Variants of K_v_4.3 Alter I_Na_ from Na_v_1.5 and Na_v_1.1

**Figure 2:**
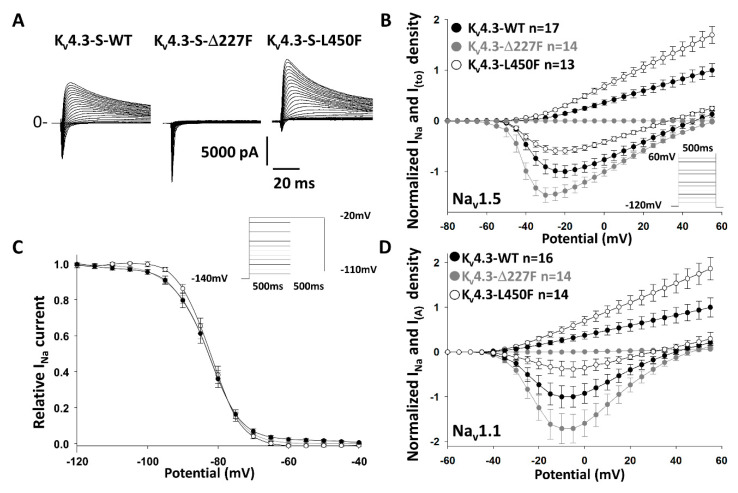
K_V_4.3 variants affect I_Na_. (**A**): Representative traces of I_to_ and I_Na_ measured in HEK293 cells co-expressing the bicistronic construct Na_v_1.5/K_v_4.3-WT, -Δ227F or -L450F (pKCND3-Short-poliovirus-SCN5A) with Na_v_β1/GFP reporter gene (pGFP-IRES-SCN1B). (**B**): Normalized current–voltage relationships, the trafficking-efficient L450F leads to an increase of I_to_ and a significant decrease in I_Na_ at potentials positive to −35 mV *p* = 0.039 (*p* < 0.001 at −20 mV), whereas the trafficking-deficient Δ227F-K_v_4.3 leads to a decrease of I_to_ but a significant increase in I_Na_ at potentials positive to −45 mV *p* = 0.018 (*p* < 0.001 at −20 mV). (**C**): Steady state inactivation of I_Na._ K_v_4.3 variants did not affect I_Na_ steady-state inactivation. (**D**): Normalized current–voltage relationship in HEK293 cells stably expressing Na_v_1.1, Na_v_β1 and Na_v_β2 and transfected with K_v_4.3-S WT vs mutants (pGFP-IRES-KCND3-short). The trafficking-efficient L450F leads to an increase of I_to_ but a significant decrease in I_Na_ at potentials positive to −30 mV *p* = 0.04 (*p* < 0.001 at −5 mV), whereas the trafficking-deficient Δ227F-K_v_4.3 leads to a decrease of I_to_ but a significant increase in I_Na_ at potentials positive to −20 mV *p* = 0.05 (*p* < 0.001 at −5 mV). Note: *n* represents the number of cells recorded.

In another series of experiments, we sought to determine whether alterations in the expression of K_v_4.3 channels can affect I_Na_. We measured I_Na_ in cells expressing the LOF trafficking-deficient channel Δ227F-K_v_4.3, the GOF trafficking efficient L450F-K_v_4.3 and WT-K_v_4.3. To avoid problems of transfection of multiple plasmids, we engineered a bicistronic construct, p*KCND3*-*SCN5A*. Thus, we co-transfected the pGFP-*SCN1B* with our WT or variant p*KCND3-SCN5A* bicistronic constructs. The positive green cells displaying the two currents therefore contained all three genes. Interestingly, even in presence of Na_v_β1, the trafficking-deficient K_v_4.3-Δ227F significantly increased I_Na_ compared to WT and the GOF K_v_4.3-L450F channel led to a significant decrease in I_Na_ when compared to WT and K_v_4.3-Δ227F channels (Figure 2A,B; Supplementary Figure S4). Steady-state inactivation of I_Na_ was not significantly affected by the trafficking-efficient or -deficient K_v_4.3 channels (Figure 2C). These results strongly support the hypothesis that the ability of K_v_4.3 to traffic or not can regulate Na_v_1.5 function and likely its trafficking, even in the presence of Na_v_β1. In order to assess whether other voltage-gated sodium channel family members can be similarly affected, we performed experiments in a different model, in which we transfected WT or variant K_v_4.3 channels into an HEK293 cell line stably expressing Na_v_1.1, Na_v_β1 and Na_v_β2. Very similar results were obtained; I_Na_ was significantly decreased in cells expressing the trafficking efficient K_v_4.3-L450F channel and significantly increased in cells expressing the trafficking deficient K_v_4.3-Δ227F channel compared to cells expressing the K_v_4.3-WT channel. To exclude a potential effect of an overlap between I_Na_ and I_to_, we designed a protocol allowing us to record I_Na_ free of the influence of I_to_ as explained in the Methods section. Under these conditions, I_to_ remains at the closed state, while I_Na_ recovers by 83.3 ± 0.02% (Supplementary Figure S5). Interestingly, we show that with I_to_ inactivated, I_Na_ recorded from cells expressing K_v_4.3-L450F remains significantly reduced compared to those expressing K_v_4.3-WT (Supplementary Figure S6). This control experiment excludes a potential overlap between inward and outward current as the cause of the significant decrease of I_Na_ in presence of a larger I_to_.

### 2.3. Beta-Subunits of the Megacomplex Regulate the Balance between I_Na_ and I_to_

**Figure 3:**
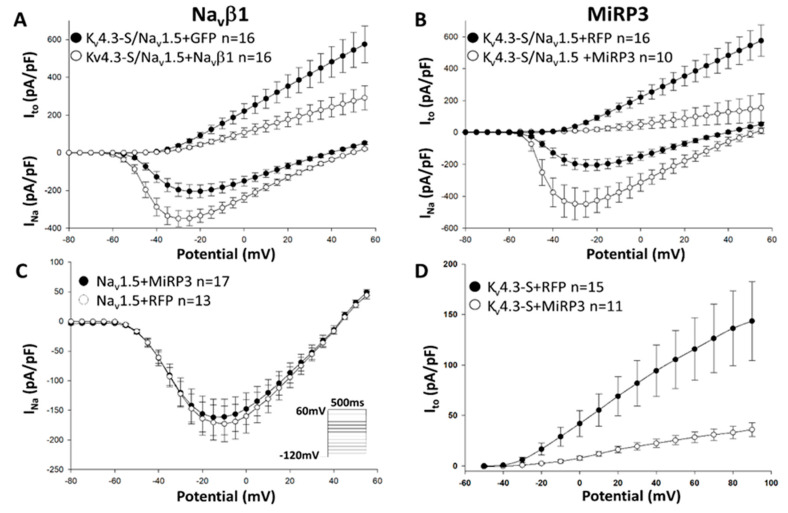
Na_v_β1 and MiRP3 decrease I_to_ and increase I_Na_. HEK293 cells were transfected with the bicistronic construct Na_v_1.5/K_v_4.3 (pKCND3-Short-poliovirus-SCN5A), with or without Na_V_β1 (pGFP-IRES-SCN1B vs pGFP) or MiRP3 (pRFP-IRES-KCNE4 vs pRFP) (**A**,**B**), or with Na_v_1.5 (pcDNA3.1-GFP-SCN5A)(C), or K_v_4.3 (pGFP-IRES-KCND3-Short)(D) with MiRP3. (**A**): Current–voltage relationship measured in HEK293 cells co-expressing the bicistronic construct Na_v_1.5/K_v_4.3 (pKCND3-Short-poliovirus-SCN5A), with or without Na_V_β1 (pGFP-IRES-SCN1B vs pGFP) showing significant I_Na_ increase in presence of Na_v_β1 at potentials positive to −45 mV *p* < 0.001 (*p* < 0.001 at −20 mV) and I_to_ decrease in presence of Na_v_β1 at potentials positive to −5 mV *p* = 0.049 (*p* < 0.001 at +40 mV) (**B**): I_Na_ increases significantly in presence of MiRP3 at potentials positive to −45 mV *p* < 0.001 (*p* < 0.001 at −40 mV) while I_to_ decreases significantly at potentials positive to −10 mV *p* = 0.039 (*p* < 0.001 at +40 mV). (**C**): In absence of K_v_4.3, MiRP3 has no effect on I_Na_. (**D**): In absence of Na_v_1.5, MiRP3 decrease I_to_ at potentials positive to −20 mV *p* < 0.001 (*p* < 0.001 at +40 mV) Note: β-subunits that decrease I_to_ lead to a significant increase of I_Na_ only if both channels, Na_v_1.5 and K_v_4.3, are present. *n* represents the number of cells recorded.

To better understand how the interacting proteins of the megacomplex modulate the balance between I_Na_ and I_to_, we co-expressed the bicistronic construct with several beta-subunits: Na_v_β1 encoded by *SCN1B*, MiRP3 encoded by *KCNE4* or KChIP2 encoded by *KCNIP2*. Co-transfection of *SCN1B* with the bicistronic construct yielded an increase of I_Na_ and a significant decrease in I_to_ (Figure 3A; Supplement Figure S7: Raw traces of Na_v_1.5 + K_v_4.3 in presence of β-subunits). Co-expression of *MiRP3*, which is known to reduce the trafficking of K_v_4.3 channels and therefore I_to,_ significantly increased I_Na_ (Figure 3B; Supplementary Figure S7). Of note, in control experiments, the co-expression of MiRP3 with Na_v_1.5 in the absence of K_v_4.3, did not modify I_Na_ (Figure 3C), while a drastic reduction of I_to_ was observed in cells expressing only K_v_4.3, as expected (Figure 3D).

**Figure 4:**
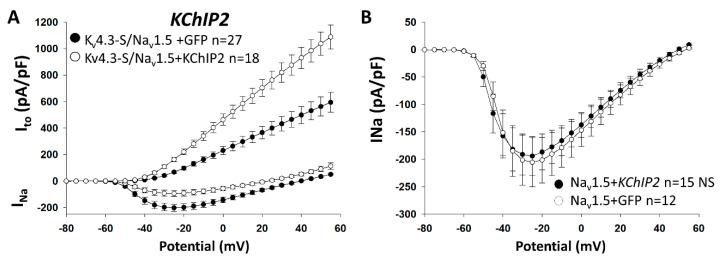
KChIP2 known to increase I_to_ decrease I_Na_. HEK293 cells were transfected with either the bicistronic construct Na_v_1.5/K_v_4.3 (pKCND3-Short-poliovirus-SCN5A) (A, C), or with Na_v_1.5 (pGFP-SCN5A) (B), with or without KChIP2 (pGFP-KCNIP2 vs pGFP), Na_v_β1 (pGFP-IRES-SCN1B vs pGFP), or MiRP3 (pRFP-IRES-KCNE4 vs pRFP). (**A**): Current–voltage relationships show that I_Na_ is significantly decreased in the presence of KChIP2 at potentials positive to −45 mV *p* = 0.024 (*p* = 0.002 at −40 mV), whereas I_to_ is significantly increased at potentials positive to −40 mV *p* = 0.009 (*p* < 0.001 at +45 mV). (**B**): In absence of K_v_4.3, KChIP2 has no effect on I_Na_. *n* represents the number of cells recorded.

Similarly, when the bicistronic construct K_v_4.3/Na_v_1.5 was expressed with KChIP2 the expected increase of I_to_ led to a drastic decrease in I_Na_ (Figure 4A; Supplementary Figure S7). In our control experiments co-expression of KChIP2 and Na_v_1.5, without K_v_4.3 did not alter I_Na_ (Figure 4B). This result, once again strongly supports that the presence of K_v_4.3 is required to modulate I_Na_. In order to ensure that the presence of I_Na_ or I_to_ do not affect each other we calculated the significance of current density of I_Na_ at −40 mV (I_to_~0) along with I_to_ at +45 mV (closest to I_Na_ reversal potential, I_Na_~0). Surprisingly, we found that the beta-subunits, MiRP3 and Na_v_β1, which reduce K_v_4.3 cell surface expression, increased I_Na_. Moreover, KChIP2, which is known to increase K_v_4.3 cell surface expression, drastically reduced I_Na_ (Figure 4A); Supplementary Figure S7). These findings provide compelling evidence in support of the hypothesis that changes in I_Na_ are mediated by the presence of K_v_4.3 channels.

### 2.4. Na_v_1.5 and K_v_4.3 Are Able to Interact

**Figure 5:**
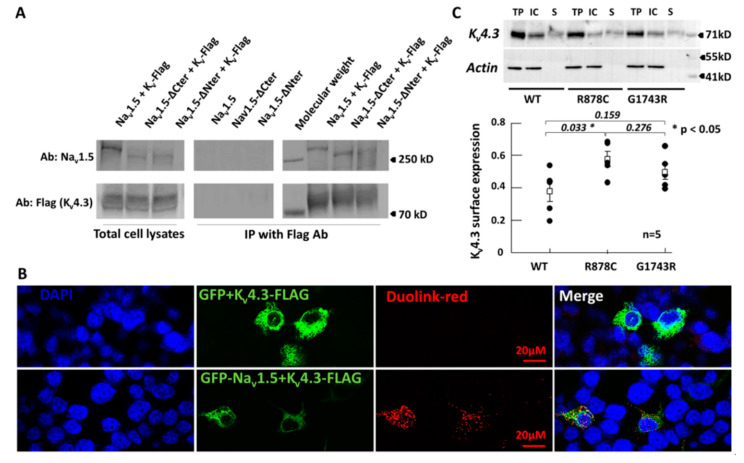
Na_v_1.5 and K_v_4.3 interact with each other. (**A**): Co-immunoprecipitation between Na_v_1.5 and K_v_4.3. HEK293 cells were transfected with WT or truncated Na_v_1.5 constructs and K_v_4.3 (pcDNA3.1-GFP-SCN5A and pCMV-KCND3-Long-Flag) as indicated above the lanes. The total cell lysates were immunoprecipitated with an anti-Flag antibody, specific to K_v_4.3-Flag, cross-linked to beads. The blots were hybridized with an anti-Na_v_1.5 antibody (top gels: Blot Ab: Na_v_1.5) or an anti-Flag antibody (bottom gels: Blot Ab: Flag). The left side corresponds to the total cell lysates of transfected cells before IP. The right side (IP with Flag Ab) corresponds to the elution fractions from beads. The negative control (center of panel A), consisting in an anti-Flag immunoprecipitation in lysates of cells expressing only GFP-Na_v_1.5 channels, clearly excluded any non-specific interaction between Na_v_1.5 and K_v_4.3 channels. The results demonstrated an interaction between K_v_4.3 and Na_v_1.5 (*n* = 7). (**B**): Duolink between GFP-Na_v_1.5 and K_v_4.3-Flag. The top line corresponds to cells co-expressing GFP alone (pGFP) with K_v_4.3-Flag while the bottom line cells co-expressing GFP-Na_v_1.5 with K_v_4.3-Flag. Only cells expressing GFP-Na_v_1.5 and K_v_4.3-Flag display red positive signals indicating a close proximity between the two channels. Note: Close proximity of the two channels can be observed within intracellular compartments. (**C**)**:** Cell surface biotinylation of K_v_4.3 in presence of WT, R878C or G1743R GFP-Na_v_1.5 channels. TP: Total Protein, IC: Intracellular, S: Surface. Note that values of S abundance were not directly quantitated from blots, but calculated as detailed in the method section. The presence of R878C significantly increases the cell surface expression of K_v_4.3, * *p* = 0.033 consistent with the observed increase of I_to_. Note: In cells co-expressing Na_v_1.5 and K_v_4.3, the two channels were co-immunoprecipitated.

In order to determine whether the inter-regulation between Na_v_1.5 and K_v_4.3 channels could be due to an interaction, we performed co-immunoprecipitation assays after co-transfection with tagged-channels (GFP-Na_v_1.5 and K_v_4.3-Flag) in HEK293 cells. A positive signal for co-immunoprecipitation was observed for both K_v_4.3 and Na_v_1.5 channels (Figure 5A and Supplementary Figure S8). It is noteworthy that Na_v_1.5-ΔCter (missing the cytoplasmic end of the protein) and Na_v_1.5-ΔNter (missing the cytoplasmic N-terminus) still co-immunoprecipitated with K_v_4.3 (Figure 5A and Supplementary Figure S8). Moreover, no signal was detected when immunoprecipitation was performed using the anti-Flag antibody (specific to K_v_4.3-Flag) on cell lysates expressing only Na_v_1.5 channel constructs as a negative control. This indicates that immunoprecipitation of Na_v_1.5 is conditioned and specific to the presence of K_v_4.3. Taken together, these assays demonstrate that the two channels are able to interact without involvement of the Na_v_1.5 N- or C-termini. In order to further investigate a potential interaction in living cells, we performed the Duolink technique enabling the visualization of proteins in close proximity in situ. We observed that cells co-expressing both GFP-Na_v_1.5 and K_v_4.3-Flag channels show robust positive red signals (Figure 5B). In contrast, cells co-expressing only GFP and K_v_4.3-Flag, used as negative controls, did not display any red signal, discounting nonspecific interaction between GFP and the K_v_4.3 channels. Moreover, this experiment allowed us to visualize that Na_v_1.5 and K_v_4.3 reside in close proximity (<40 nm) at the membrane but also within intracellular compartments, supporting the hypothesis suggesting trafficking as one of the potential mechanisms regulating the I_Na_/I_to_ balance.

Additionally, cell surface biotinylation revealed that the Na_v_1.5-R878C variant enhances cell surface expression of K_v_4.3 channels (Figure 5C and Supplementary Figure S9), in agreement with our data showing an increase of I_to_ in the presence of the Na_v_1.5 variant (Figure 1). It is noteworthy that cell surface expression of K_v_4.3 channels was not significantly different in the presence of the trafficking deficient G1743R-Na_v_1.5 compared to WT-Na_v_1.5 (Figure 5B). This result is also in agreement with our electrophysiology recordings showing that the 62.9% loss of peak I_to_ is due to the shift of the steady state inactivation in presence of the −40 mV prepulse. Indeed, in the protocol depicted in Figure 1, a prepulse at −40 mV was used to inactivate the WT sodium channel. This prepulse led to inactivation of a much larger portion of K_v_4.3 channel in presence of Na_v_1.5-G1743R compared to Na_v_1.5-WT, as a result of the steady-state inactivation shift (Figure 1C). Indeed, at −40 mV, 49 ± 0.03% K_v_4.3 channels are inactivated in presence of G1743R-Na_v_1.5 compared to WT-Na_v_1.5 consistent with the loss of function recorded in Figure 1A.

## 3. Discussion

The voltage-gated sodium channels Na_v_1.5, responsible for I_Na_, play a crucial role in excitability and impulse propagation in the heart. K_v_4.3 channels are responsible for I_to_ which gives rise to phase 1 of the cardiac AP. A fine balance between depolarization and repolarization during the early phase of the AP regulates action potential characteristics. An imbalance between the two currents, in particular a loss of function of I_Na_ and/or a gain of function in I_to,_ can importantly accentuate the AP notch leading to accentuation of the electrocardiographic J wave. Amplification of the J wave often appears as an ST segment elevation in the ECG and can predispose to the development of BrS and/or early repolarization syndrome, which comprise the J-wave syndrome [1,12,13,14,15]. Na_v_1.5 and K_v_4.3 α-subunits have always been considered to be functionally independent. However, an increasing body of evidence points to the fact that voltage-gated ion channel α-subunits may not function completely independently of each other [2,10,11,16,17,18].The results described in the present study point to a fundamentally different model of sodium and potassium α-subunits interaction and function. We demonstrate that Na_v_1.5 and K_v_4.3 α-subunits interact and inter-regulate each other’s function.

Our previous work has shown that voltage-gated sodium α-subunits are able to interact and form functional dimers mediated through recruitment of 14-3-3 proteins regulating the coupled gating of voltage-gated sodium channels responsible for the rapid upstroke of the AP in excitable tissues [2,10,11]. Thus, alteration of trafficking and gating of pathological channels can result in dominant-negative suppression leading to BrS [2,11,19]. We further showed that Na_v_1.1 and Na_v_1.2 are able to dimerize, which has far-reaching implications in neurological disorders including epilepsy or spinocerebellar ataxia [10,11,20]. Matamoros, et al. demonstrated that Kir2.1 and Na_v_1.5 α-subunits interact via α-syntrophin [17]. This interaction modulates the balance between I_K1_ and I_Na_ and supports the concept and importance of exploring the intricacies of megacomplex formation [17]. Their subsequent study demonstrated that Kir2.1 and Na_v_1.5 share a common pathway of trafficking to the cell surface, thus influencing cell excitability [18]. Consequently, disruption of Kir2.1 trafficking in cardiomyocytes affects trafficking of Na_v_1.5, which has important implications in the development of arrhythmias associated with inherited cardiac diseases. A recent study also reports interaction [2,11,19] between K_v_4.3 and K_v_11.1 (or hERG) proteins, leading to an increase in I_Kr_ current density when K_v_11.1 and K_v_4.3 are co-expressed [21]. Finally, Bilicki et al. have shown that a K_v_7.1 trafficking-deficient variant impairs cell surface expression of K_v_11.1 by physical interaction of the α-subunits responsible for I_Kr_ and I_Ks_ [22]. The list of auxiliary proteins interacting and regulating the trafficking and the gating of both Na_v_1.5 and K_v_4.3 is ever-increasing. Na_v_β1 and SAP97 have previously been identified as important modulators. Recent work from Belau et al., demonstrated that DPP10 a previously known regulator of K_v_4.3, also regulates the trafficking and gating of Na_v_1.5 [23]. At last, in support of our hypothesis, Portero et al. recently showed that an increased expression of K_v_4.3 could lead to a decrease in I_Na_ [4].

**Figure 6:**
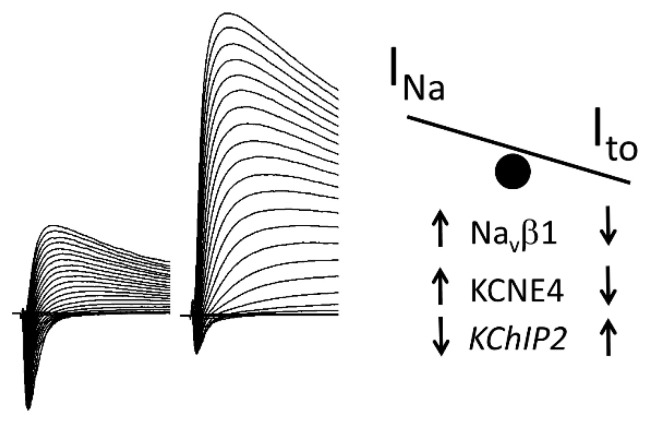
Schematic of the influence of β-subunits of the two channels on the I_Na_/I_to_ balance. Left panel represents raw traces of I_Na_/I_to_ displaying a larger I_Na_ and smaller I_to_ as opposed to a smaller I_Na_ and larger I_to_ that could potentially lead to the expression of BrS. The right schematic shows a schematic of the influence of the β-subunits of the two channels on the I_Na_/I_to_ balance.

In the present study, we investigated the potential for Na_v_1.5 variants to alter I_to_ and K_v_4.3 variants to affect I_Na_. We showed that contrary to traditional belief, K_v_4.3 and Na_v_1.5 do not function independently and that they are able to inter-regulate each other, thus modulating their respective trafficking and gating. We were able to show that impairment of K_v_4.3 trafficking, secondary to expression of *KCNE4*, leads to an increase of I_Na_. In contrast, increased expression of *KChIP2* produced an increase in I_to_ as well as a decrease in I_Na_. A representation of this mechanism is schematically represented Figure 6. The J wave or ST segment elevation associated with BrS is known to be more prominent in the right ventricle (RV), accounting for the right ventricular nature of the syndrome. It has long been appreciated that this is due to the presence of a prominent AP notch in right versus left ventricle (LV), which in turn is due to the presence of a more prominent I_to_ in RV versus LV [24]. In support of our hypothesis, a recent study reported that, in addition to a more prominent I_to_, RV displays a less prominent I_Na_ than LV, thus contributing to the appearance of a more prominent notch and J wave in RV [25]. 

Our study shows that *SCN5A* LOF variants can alter I_to_ by modulating K_v_4.3 cell surface expression (R878C-Na_v_1.5) or by shifting its steady state inactivation (G1743R-Na_v_1.5). Reciprocally, the *KCND3* GOF and LOF variants, L450F- and Δ227F-K_v_4.3, are capable of decreasing and increasing I_Na_ from both Na_v_1.5 and Na_v_1.1 channels respectively, strongly suggesting that similar mechanisms of regulation are present in the heart and in the brain. We then sought to investigate whether the nonsense variant E555X could also alter I_to_. Interestingly, the E555X variant was originally uncovered in a young child with BrS. Park et al. generated a pig model of this variant (E558X) in an attempt to recapitulate the Brugada phenotype in the pig [26]. Unlike in humans, in the pig this mutation led to cardiac conduction defect rather than BrS, with no hint of an ST-segment elevation or J wave in the ECG, even when challenged with the sodium channel blocker flecainide [26]. This was not surprising given that K_v_4.3 is not expressed in the pig ventricle, which lacks I_to_ [1]. This finding, together with those reported by others [13,27,28] strongly support that the presence of a prominent I_to_ would lead to the expression of BrS/ERS as opposed to only cardiac conduction defect as a consequence of Na_v_1.5 LOF mutations. The present study provides further evidence in support of this hypothesis, demonstrating a significant increase in I_to_ by this nonsense mutation that causes total loss of I_Na_. The effect of these genetic variants to produce reciprocal modulation of K_v_4.3 and Na_v_1.5 ion channel activity leads to a synergistic shift in the balance of currents in the early phases of the RV epicardial action potential, thus accentuating the J wave or ST segment elevation in the ECG and the BrS phenotype. Inter-regulation of sodium channels with K_v_4.3 pathogenic variants also provides new insights into factors contributing to potential mechanisms underlying the expression of spinocerebellar ataxia. Autosomal dominant cerebellar ataxias (SCAs) are progressive neurodegenerative disorders resulting from atrophy of the cerebellum leading to progressive ataxia of gait and limbs, as well as speech and eye movement difficulties [29]. Intriguingly, some SCAs and BrS possess striking similarities. They can be both associated with a loss of function of I_Na_ or a gain of function of K_v_4.3 channels. Indeed, we previously demonstrated a key role for the N-terminus of Na_v_1.5 in channel trafficking [2], since variants in this region lead to retention of the variant in the endoplasmic reticulum and to BrS [2]. Likewise, Sharkey et al. reported a similar trafficking defect for Na_v_1.6 (*SCN8A*) N-terminal variants associated with ataxia [30]. These authors demonstrated that the channel is retained in the cis-Golgi resulting in reduced levels of Na_v_1.6 at the nodes of Ranvier in vivo. To date, 22 causal genes have been associated with spinocerebellar ataxia [29,31]. Twelve variants in *KCND3* have been associated with spinocerebellar ataxia (SCA19/22). Ten of these variants have been shown to cause a LOF and two a GOF of I_A_ [5,7,32,33,34,35,36,37]. However, the mechanism whereby K_v_4.3 GOF mutation lead to SCA19/22 remain ununderstood. Interestingly, the two GOF variants, p.L450F and p.G600R located in the C-terminus of the channel have also been previously reported to be associated with BrS. Moreover, a missense variant in the C-terminus, p.R431C, was linked to episodic ataxia [38] and a de novo duplication of *KCND3* was reported to cause early repolarization syndrome [39]. Furthermore, Takayama et al. recently reported the K_v_4.3 GOF variant, p.G306A, to be responsible for early repolarization syndrome, refractory epilepsy, intellectual disability and paroxysmal atrial fibrillation [40]. Collectively, these studies provide further support for the hypothesis that GOF variants in *KCND3* may share a common pathway to cardiac and neuronal channelopathies sometimes in the same patient.

The L450F variant of *KCND3* studied in the present study has been associated with both BrS and SCA19/22 [3,41]. The L450F-K_v_4.3 mediated GOF in I_to_ in the heart is consistent with the ionic mechanisms causing BrS. Although a similar GOF in I_(A)_ was reported to cause spinocerebellar ataxia SCA19/22, the specific disease mechanism remains to be fully elucidated. Our findings suggest that the K_v_4.3 GOF variants may give rise to ataxia due to a significant decrease of I_Na_ rather than an increase of I_(A)_ [5]. This hypothesis remains to be more fully tested. 

Until recently, studies of *SCN5A* or *KCND3* variants have generally been approached with the traditional view that they are likely to exert an influence on I_Na_ or I_to_, exclusively. Our findings highlight the need to expand our view of the megacomplex, also termed “the channelosome” to include association of Na_v_1.5 and K_v_4.3 α-subunits regulating each other’s trafficking and gating. It is not yet known whether interaction between Na_v_1.5 and other potassium channels such as Kir2.X and K_v_4.3 occurs in the same mega-complex and how their interaction impacts the dimerization of sodium channels. We could speculate that interactions between different α-subunits could also regulate the subcellular location of the channelosome, e.g., intercalated discs *vs* lateral membrane in cardiac myocytes or axon *vs* initial segment in neurons. Indeed, it was shown that distinct pools of Na_v_1.5 channels are directed either toward the lateral membrane as opposed to intercalated discs depending on whether they interact with α-syntrophin or SAP97 [42,43,44]. Our observations provide new insights into a wide range of cardiac and non-cardiac channelopathies, including epilepsy and spinocerebellar ataxia. The resulting paradigm shift is likely to open new perspectives for genetic screening of cardiac arrhythmia and other channelopathies caused by *SCNXA* or *KCND3* variants. The knowledge gained may also be helpful in the design of novel approaches to therapy for these allelic syndromes.

## 4. Methods 

### 4.1. cDNA Cloning and Mutagenesis

The following plasmids, all containing human channel subunit sequences, were used in this study: pcDNA3.1-GFP-SCN5A [2], pcDNA3.1-GFP-SCN5A-L1821fsX10 (= ΔCter), pGFP-N3-SCN5A-ΔNter generated by truncation of the 381 first nucleotides of SCN5A (127 amino acids), and replacement of residue 128 by a methionine (= ΔNter) [2], pGFP-poliovirus-SCN5A [11], pGFP-IRES-KCND3-Short and pCMV-KCND3-Long-FLAG. The bicistronic construct pKCND3-short-poliovirus-SCN5A plasmid was performed by Genscript (Piscataway, NJ, USA), SCN5A and KCND3 genes were subcloned and inserted into the pGFP-IRES plasmid by substituting GFP by KCND3-short. The pGFP-IRES-SCN1B, pDS-RED-IRES-KCNE4, pDS-RED-IRES, pGFP-IRES-KCNIP2 and pGFP-IRES were used to study the effect of the beta subunits with the bicistronic plasmid. Their coding sequences were CCDS844.1 for the-short isoform of *KCND3* and CCDS843.1 for the long, CCDS 2456.2 for *KCNE4,* CCDS41562.1 for *KCNIP2,* and CCDS 46047.1 for *SCN1B.* Variants were prepared using the QuikChange II XL Site-Directed Mutagenesis Kit (Agilent Technologies/Stratagene, Santa Clara, CA, USA) according to the manufacturer’s instructions and verified by sequencing. 

### 4.2. HEK293 Cell Culture and Transfection

HEK293 cells were maintained in DMEM supplemented with 10% heat-inactivated fetal calf serum and 1% penicillin/streptomycin. For patch-clamp recordings, transfections were done with Polyfect transfection (Qiagen, Germantown, MD, USA) according to the manufacturer’s instructions and cells were transfected with the constructs of interest in 35-mm well dish, with a total of 0.6 µg of pCDNA3.1-GFP-*SCN5A* and 0.3 µg of *KCND3* construct, molar ratio 2 to 1. Experiments using the bicistronic construct used 1.0 µg of bicistronic construct and 0.5 µg of the Beta IRES GFP subunit of interest in a ratio 2 to 1, per 35 mm dish. HEK293 Na_v_1.1, Na_v_β1, Na_v_β2 stable cell lines were a generous gift of Dr. Alfred George, Jr. from Northwestern University, Chicago, IL.

For co-immunoprecipitation experiments, cells were transfected with 1.5 µg of each channel plasmid per 25-cm^2^-culture flask using jetPEI (Polyplus Transfection, New York, NY, USA), except for negative controls using cells expressing only Na_v_1.5 constructs. All experimental data provided were performed with a minimum of 3 independent transfections; the *n* in electrophysiology figures represents the number of cells recorded.

### 4.3. Solutions for Electrophysiological Recordings

Thirty-six hours after transfection, HEK293 cells were trypsinized and seeded to a density that enabled single cells to be identified. Green positive cells were chosen for patch-clamp experiments. For patch clamp recordings, cells were bathed in an extracellular Tyrode’s solution containing in mM: 150 NaCl, 2 KCl, 1 MgCl_2_, 1.5 CaCl_2_, 1 NaH_2_PO_4_, 10 glucose, 10 HEPES, pH 7.4 (NaOH). Patch pipette medium was in mM: 125 KCl, 25 KOH, 1 CaCl_2_, 2 MgCl_2_, 4 K-ATP, 10 EGTA, 10 HEPES, adjusted to pH 7.2 with KOH. For I_Na_ recording only in Supplemental Data Figure S1, cells were bathed in an extracellular Tyrode solution containing in mM: 135 NaCl, 4 KCL, 2 MgCl_2_, 2.5 CaCl_2_, 1 NaH_2_PO_4_, 20 glucose, 10 HEPES, adjusted to pH 7.4 with NaOH. Patch pipette medium was in mM: 5 NaCl, 140 CsCl, 2 MgCl_2_, 4 Mg-ATP, 5 EGTA, 10 HEPES, adjusted to pH 4.2 with CsOH. For recording of I_to_ in Figure 3D, extracellular was in mM: 140 NaCl, 4 mM KCl, 2 CaCl_2_, 1 MgCl_2_, 5 HEPES, 10 Glucose, adjusted to pH 7.4 with NaOH. Intracellular solution was in mM: 125 KCl, 25 KOH, 1 CaCl_2_, 2 MgCl_2_, 4 K-ATP, 10 EGTA, 10 HEPES, adjusted to pH 7.2 with KOH.

### 4.4. Electrophysiological Recordings

Patch-clamp recordings were carried out in the whole-cell configuration at room temperature. Ionic currents were recorded with Axopatch 200B (Axon Instruments, San Jose, CA, USA) amplifier. Patch pipettes (Corning Kovar Sealing code 7052, WPI, Sarasota, FL, USA) had resistances of 1.2–2.5 MΩ in the whole cell configuration. Voltage errors were reduced via series resistance compensation below 5 mV. Currents were filtered at 5 kHz (−3 dB, 8-pole low-pass Bessel filter) and digitized at 30 kHz (NI PCI-6251, National Instruments, Austin, TX, USA). Data were acquired with pClamp 10 and analyzed with Clampfit (Axon Instruments, San Jose, CA, USA).

To measure peak I_Na_ or I_to_ amplitude and determine current-voltage relationships (I-V curves), currents were elicited by 500 ms-test potentials from −100 to +60 mV by increments of 5 mV from a holding potential of −120 mV. To record I_Na_ free of the influence of I_to_, we inactivated I_to_ by introducing a 500 ms prepulse at 0 mV, which activates both channels. The test was introduced following a 10 ms inter-pulse interval at −120 mV. Because I_Na_ but not I_to_ recovers from inactivation during the 10 ms interval, I_Na_ can thus be recorded in the absence of I_to_ (Supplement Data Figure S6). For determination of steady-state inactivation of I_Na,_ a holding potential of −120 mV was used and a 500 ms conditioning prepulse was applied in 5 mV increments between −140 and −30 mV, followed by a 500-ms test pulse at −20 mV. For I_to_ steady-state inactivation the conditioning prepulses were performed between −120 mV and 0 mV, followed by a 500-ms test pulse at 40 mV.

Data for the activation-V_m_ and steady-state availability-V_m_ relationships of I_Na_ were fitted to the Boltzmann equation as described in Clatot et al. [11].

### 4.5. Co-Immunoprecipitation 

Forty-eight hours after transfection with channel constructs, HEK293 cells were washed with phosphate buffer saline (PBS) and whole cell protein lysates were isolated using lysis buffer (50 mM Tris pH 7.5, 150 mM NaCl, 2 mM EDTA, 1% Triton and protease inhibitor cocktail from Sigma-Aldrich, Saint Louis, MO, USA). Cell pellets were flushed 20 times through a 25-gauge needle, rotated for 1 h at 4 °C, and finally centrifuged for 30 min at 16,000× *g*. Dynabeads Protein G (Invitrogen, Waltham, MA, USA) washed twice with PBS-tween 0.02%, were, either used to pre-clear total proteins for 1 h at room temperature, or incubated with the anti-Flag antibody (Sigma-Aldrich, Saint Louis, MO, USA) for 2 h at room temperature, washed twice again with PBS-tween 0.02%, and incubated with the pre-cleared lysates. Samples were rotated overnight at 4 °C. After washing the beads 4 times with PBS-tween 0.02%, proteins were eluted with the Laemmli sample buffer at 37 °C for 30 min under agitation, separated on a NuPAGE 7% Tris-Acetate gel (Invitrogen), transferred to a nitrocellulose membrane and incubated with primary antibodies: mouse anti-Flag (1:500, Sigma-Aldrich), rabbit anti-Na_v_1.5 antibody (1:200, Alomone Labs, Jerusalem, Israel), and mouse anti-transferrin receptor (1:500, Invitrogen) as a loading control. Bound antibodies were detected using DyLight-conjugated secondary antibodies (Thermo-Fisher, Waltham, MA, USA), and protein signals were visualized using a Li-Cor Odyssey (Li-Cor Biosciences, Lincoln, NE, USA).

### 4.6. Duolink 

The Duolink technique enables the detection and visualization of protein interactions in tissue and cell samples prepared for microscopy. Duolink detection was performed on HEK293 cell cultures fixed with methanol for 5 min at −20 °C. Cells were then washed twice for 5 min with PBS, blocked in PBS-5% BSA for 30 min at room temperature. Cells were incubated for 1 h with primary antibodies: rabbit anti-GFP (1:300, Torrey Pines Biolabs, Houston, TX, USA) against Na_v_1.5-GFP or GFP, mouse anti-Flag (1:300, Stratagene) against K_v_4.3-Flag. The pair of oligonucleotide labeled secondary antibodies (PLA probes from Millipore Sigma, Burlington, MA, USA) was used following manufacturer’s instructions and imaging was performed using confocal microscope.

### 4.7. Cell Surface Biotinylation

Surface proteins of cells on 35-mm dishes were biotinylated using a cross-linking reagent (EZ-Link Sulfo-NHS-S-S-Biotin, Pierce Biotechnology, Rockford, IL, USA). HEK293 Cells were washed three times with ice-cold PBS-CM (PBS + 1 mM MgCl_2_ and 0.1 mM CaCl2), and 1 mg/mL of biotinylation reagent in 2.5 mL of biotinylation buffer (BB in mM: 150 NaCl, 2 CaCl2, and 10 triethanolamine) or buffer alone was added for 30 min on ice. After the cells were washed with quenching buffer (PBS-CM + 192 mM glycine and 25 mM Tris at pH7.5), whole cell protein lysates were isolated using Triton lysis buffer (in mM: 150 NaCl, 1.5 MgCl2, 20 HEPES, 1% Triton X-100, and 10% glycerol, pH 7.5) with protease and phosphatase inhibitors. Biotinylated proteins were recovered from the cell lysates with prewashed streptavidin-coated agarose beads (Sigma Chemical, Rockford, IL, USA). Proteins in the biotinylated (S = cell surface) and non-biotinylated (IC = intracellular fraction) fractions along with total lysates (TL) were separated by Western blot, transferred to PVDF membranes then probed with anti-Flag (1:1000) followed by anti-actin (1:1000) antibodies. Luminescence (Clarity, BioRad, Hercules, CA, USA) was detected using a ChemiDoc scanner (BioRad) and the *Flag* signal intensity in the TL and IC fractions were quantitated using Adobe Photoshop and normalized to actin signal intensity. IC intensity was calculated as a percentage of TL intensity and S abundance determined by subtraction of the latter from 100%. 

### 4.8. Statistical Analysis

In order to test the primary hypothesis that there are significant differences between the control condition and each of the other conditions (e.g., presence of a variant or expression of beta-subunits), one-way or two-way analysis of variance (ANOVA) was used to conduct all analyses comparing control condition to each of the condition types individually with a Holm-Sidak correction, as appropriate (SigmaPlot^®^ software). Results are presented as mean ± standard error (SEMs). Significance level *p* < 0.05 was considered significant.

## Figures and Tables

**Table 1:** Electrophysiological characteristics of Ito in presence of WT and loss of function (LOF) Nav1.5 channels.

Variant	*n*	I_to_ Peak +20 mV	SEM	Fold Change	*p*	*n*	V_1/2_ SSI I_to_	SEM	Shift	*p*	Relative I_to_, HP at −40 mV	SEM	Fold Change in Peak I_to_, HP at −40 mV
WT	50	87.1	8.2	NA	NA	11	−40.03	0.93	NA	NA	48.7	0.03	1
R878C	14	243.3	57.6	2.79	<0.001	14	−39.74	1.2	0.29	NS	54.2	0.03	1.11
E555X	15	138.0	16.8	1.58	<0.001	18	−41.65	0.37	−1.62	NS	43	0.02	0.88
G1743R	18	54.8	12.9	0.63	<0.001	15	−46.22	0.75	−6.19	<0.001	32.9	0.02	0.68

## Supplementary Materials

Supplementary materials can be found at https://www.mdpi.com/1422-0067/21/14/5057/s1, Figure S1: Effect of Na_v_1.5 R878C and G1743R variants on I_Na_, Figure S2: Recovery from inactivation of K_v_4.3 in presence of SCN5A variants was not affected, Figure S3: Effect of Na_v_1.5 variants on K_v_4.3-long, Figure S4: Raw traces of Na_v_1.5 WT + Na_v_ 1 +K_v_4.3-WT or variants, Figure S5: I_Na_ recovery from inactivation was not affected by K_v_4.3 variants, Figure S6: Separating I_to_ from I_Na_ recordings to assess a potential overlap between the two currents, Figure S7: Raw traces of Na_v_1.5 + K_v_4.3 in presence of -subunits, Figure S8: Co-IP full Blot, Figure S9: Cell surface biotinylation full blots.

## Abbreviations

AP	Action Potential
BrS	Brugada syndrome
Na_v_	Sodium voltage-gated channels
I_Na_	Inward sodium current
WT	Wild type
ER	Endoplasmic reticulum
I_to_= I_A_	Transient outward potassium current
SCA19/22	Spinocerebellar Ataxia type 19 and 22
LOF	Loss of function
GOF	Gain of function
