# Supplementary material for: Inter-Regulation of Kv4.3 and Voltage-Gated Sodium Channels Underlies Predisposition to Cardiac and Neuronal Channelopathies
Source: Int J Mol Sci. 2020 Jul 17;21(14):5057. doi: 10.3390/ijms21145057 (PMC7404392; doi:10.3390/ijms21145057)
Supplement: Supplementary file 1 [file ijms-21-05057-s001.pdf]

Supplemental Figures title page:

Supplemental Figure 1: Effect of $Na_v1.5$ R878C and G1743R mutations on I_{Na}

Supplemental Figure 2: Recovery from Inactivation of $K_v4.3$ in presence of SCN5A mutants was not affected

Supplemental Figure 3: Effect of $Na_v1.5$ variants on $K_v4.3$-long

Supplemental Figure 4: Raw traces of Na^+ channels + $K_v4.3$-WT or mutants

Supplemental Figure 5: I_{Na} recovery from inactivation was not affected by $K_v4.3$ mutants

Supplemental Figure 6: Separating I_{to} from I_{Na} recordings to assess a potential overlap between the two currents.

Supplemental Figure 7: Raw traces of $Na_v1.5$+$K_v4.3$ in presence of β-subunits

Supplemental Figure 8: Co-IP full Blot

Supplemental Figure 9: Cell surface biotinylation full blots

Supplemental Figure 1: Effect of $Na_v1.5$ R878C and G1743R mutations on I_{Na}

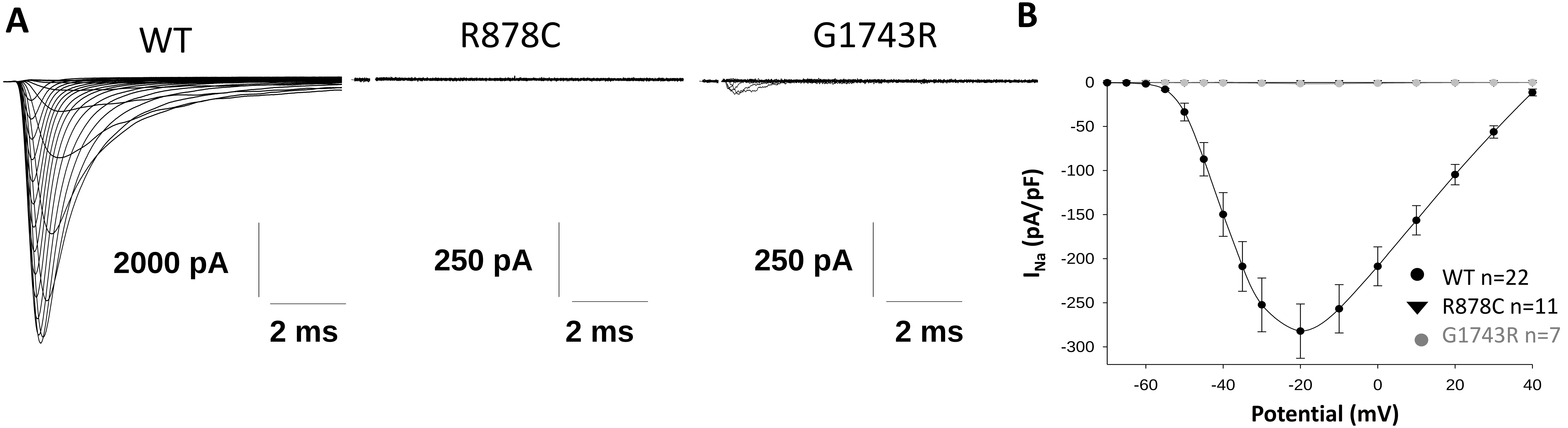

A. Representative traces of I_{Na} measured in HEK293 cells expressing the $Na_v1.5$-WT or mutants. **B.** Current-voltage relationships. *n* represents the number of cells recorded. Note: $Na_v1.5$-R878C display no-current and G1743R displays no to very little currents as previously reported by us and others Clatot et al 2012 and Valdivia . Of note E555X results in a truncated channel in the DI-DII linker and does not display any current as we reported in Clatot et al 2017.

Supplemental Figure 2: Recovery from Inactivation of $K_v4.3$ in presence of SCN5A mutants was not affected

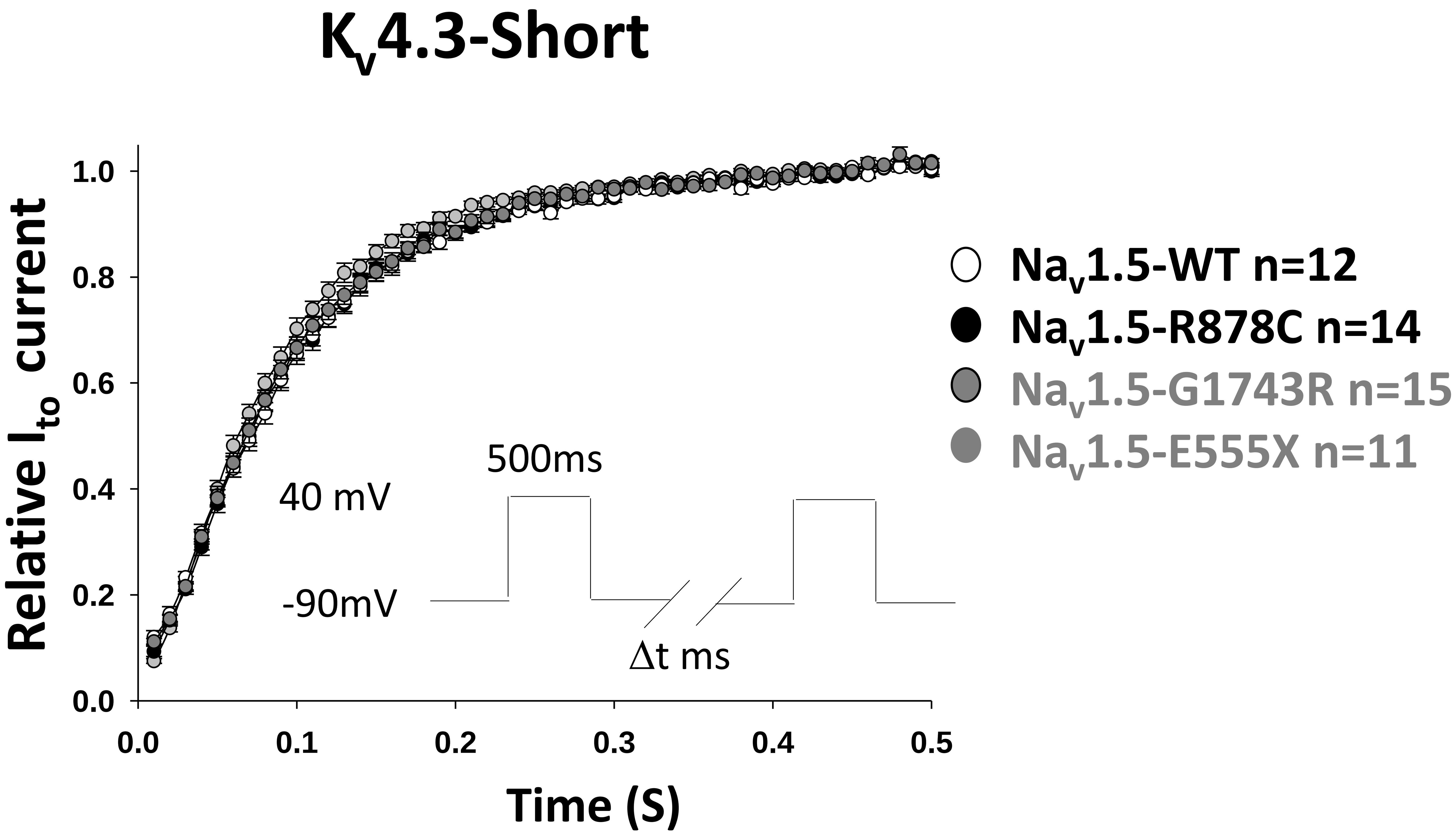

I_{to} recovery from inactivation in presence of $Na_v1.5$-WT vs mutants. **Note:** $K_v4.3$ recovery from inactivation was not altered in presence of $Na_v1.5$ mutant.

Supplemental Figure 3: Similar results were observed with K_v4.3-long

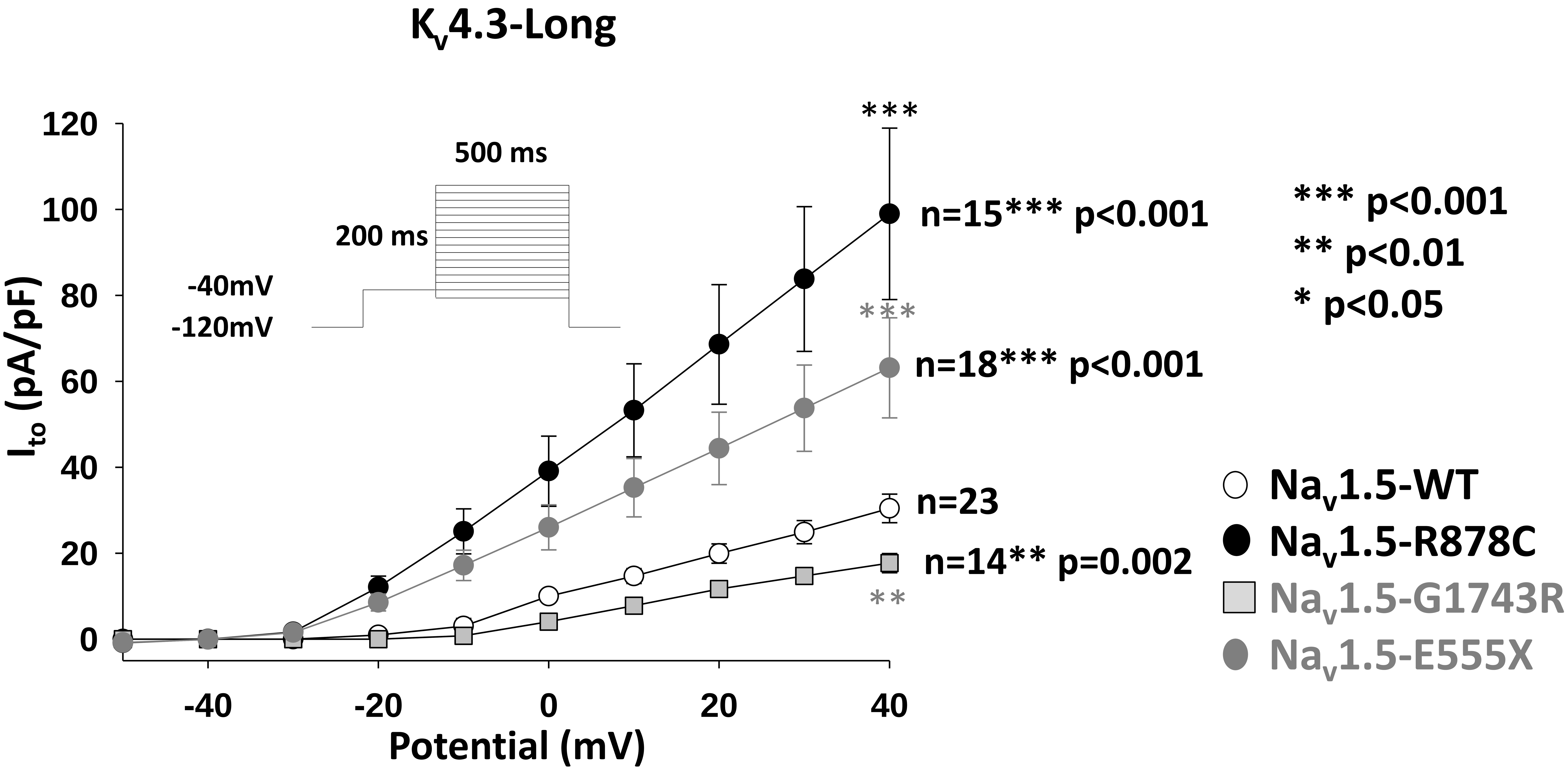

I_{to} Current-voltage relationships recorded from HEK293 cells coexpressing K_v4.3-Long (pCMV-hKCND3-Long-3FLAG) in presence of Na_v1.5-WT vs Mutants (PcDNA3.1-GFP-hSCN5A). *n* represents the number of cells recorded.

Supplemental Figure 4: Raw traces of Na^+ channels + K_v4.3-WT or mutants

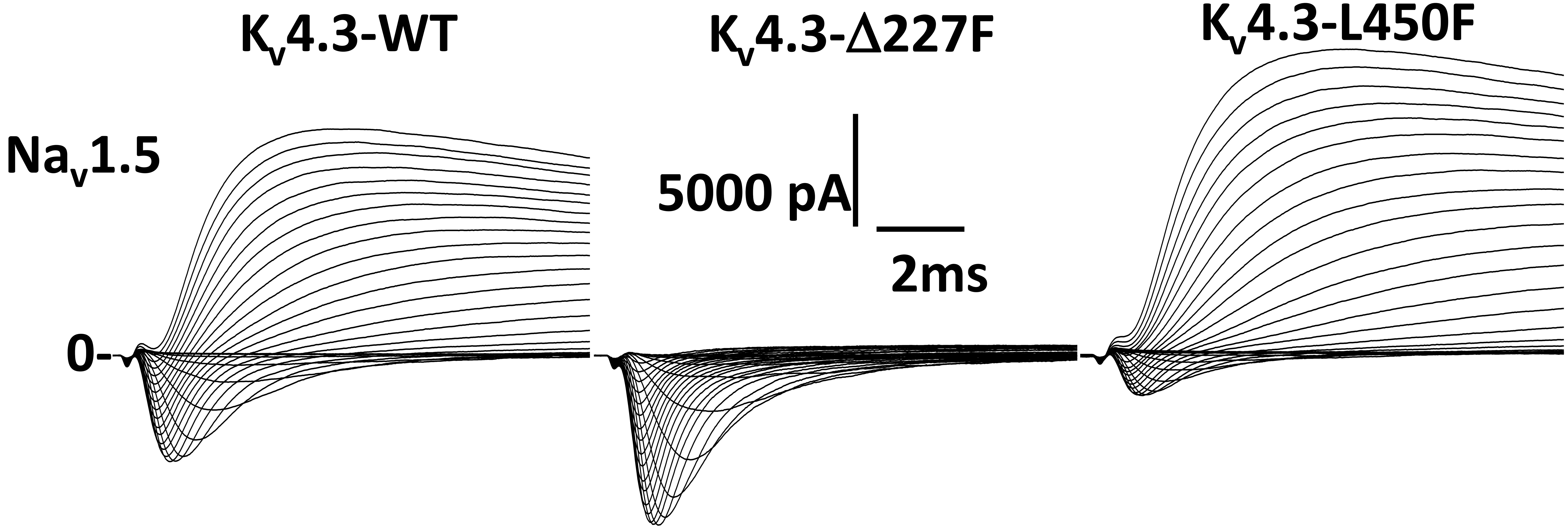

Raw traces of I_{Na}/I_{to} recorded in HEK293 cells expressing Na_v1.5, $Na_v\beta$1 with K_v4.3-WT, Δ227F or L450F.

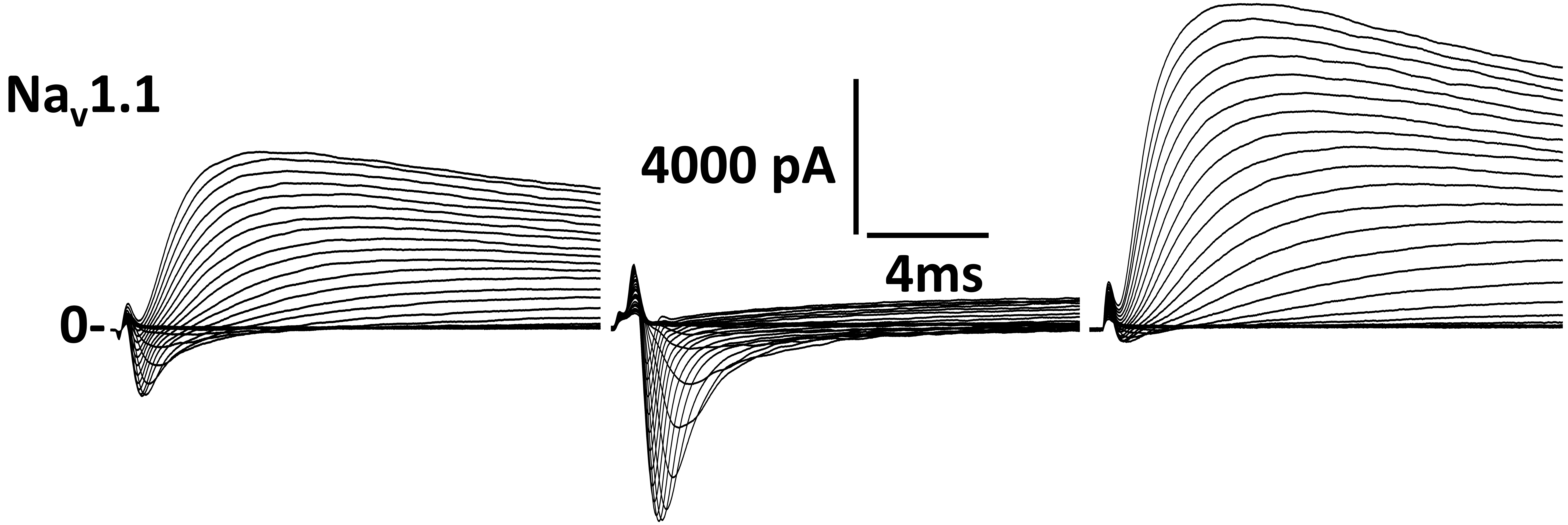

Raw traces of $I_{Na}/I_{(A)}$ recorded in HEK293 cells stably expressing Na_v1.1, $Na_v\beta$1 and $Na_v\beta$2 with K_v4.3-WT, Δ227F or L450F.

Note: Larger I_{Na} were recorded in cells expressing the LOF K_v4.3-Δ227F, compared to the reduced I_{Na} in cells expressing the GOF L450F mutant.

Supplemental Figure 5: I_{Na} recovery from inactivation was not affected by K_v4.3 mutants

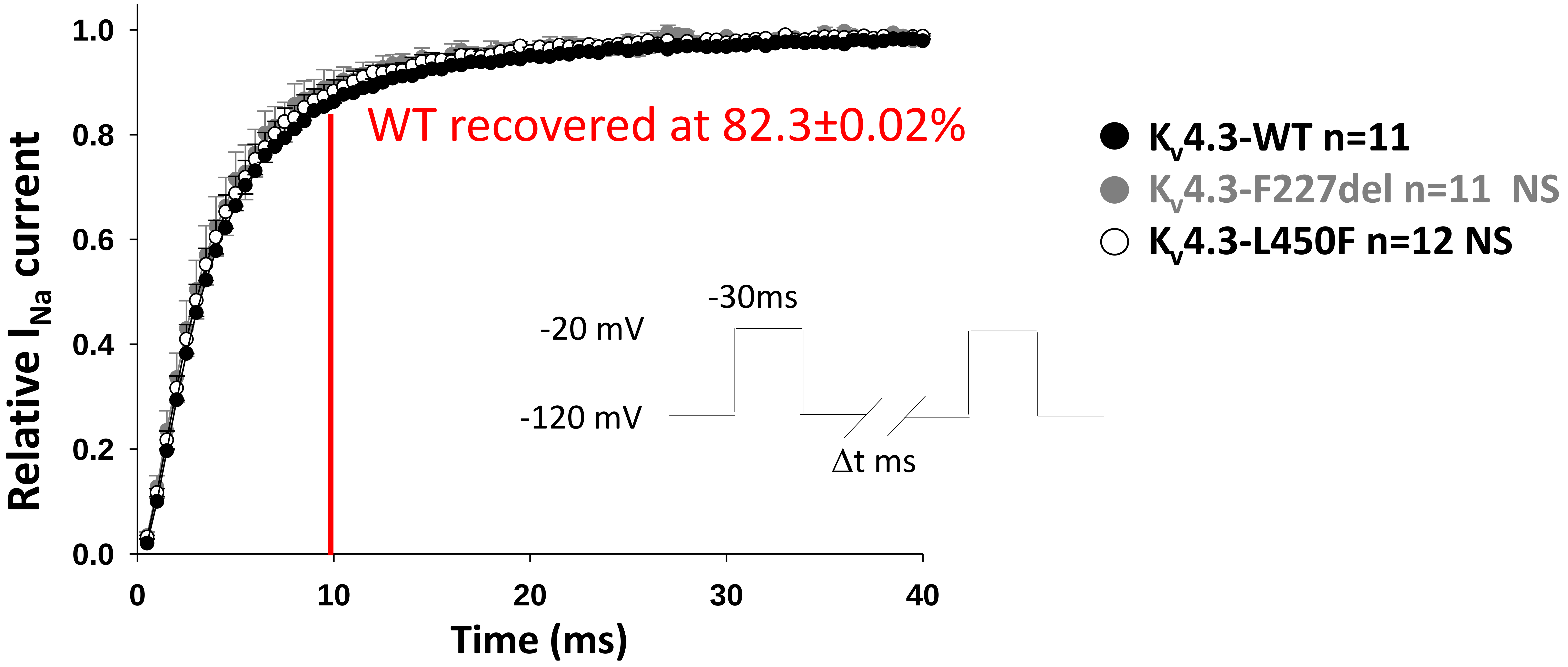

I_{Na} recovery from inactivation in presence of K_v4.3-WT vs mutants. **Note:** Na_v1.5 recovery from inactivation was not altered in presence of K_v4.3 mutants.

Supplemental Figure 6: Separating I_{to} from I_{Na} recordings to assess a potential overlap between the two currents.

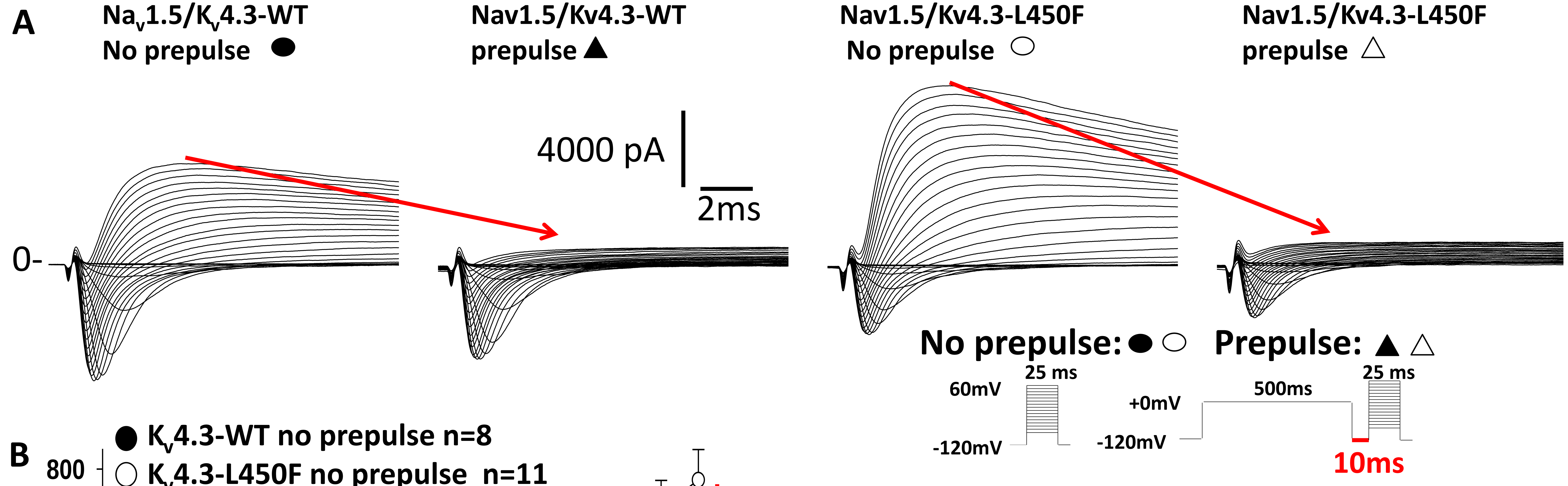

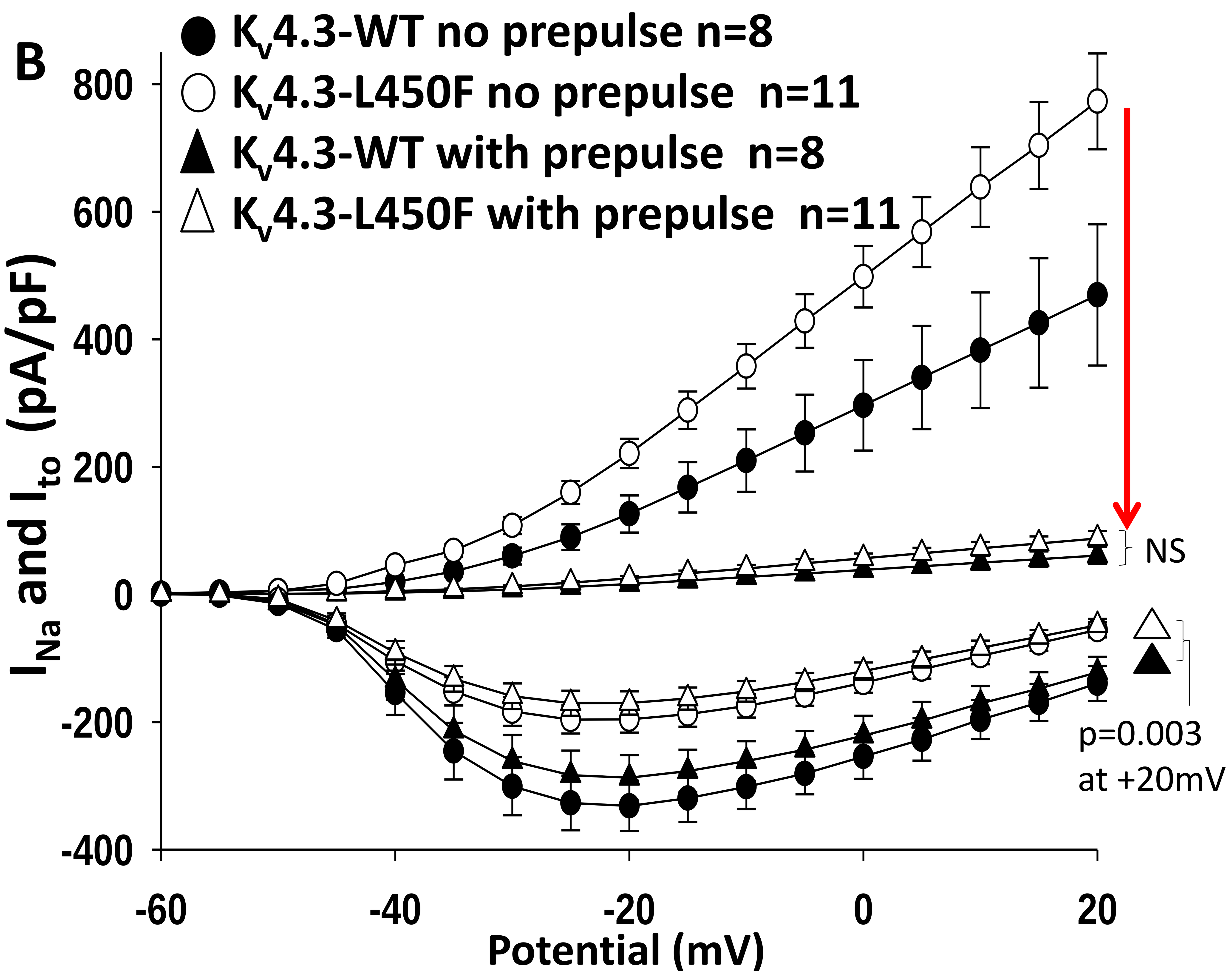

Figure: Separating I_{to} from I_{Na} recordings to assess a potential overlap between the two currents.

We designed a protocol as depicted above and described in the method section which allow to remove I_{to} from the recordings. Interestingly, we show that the inhibition of I_{to} did not significantly affect I_{Na} and failed to rescue I_{Na} in presence of the K_v4.3-L450F.

A. Representative raw traces of I_{to}/I_{Na} measured in HEK293 cells with K_v4.3-WT or L450F, with and without prepulse. **B.** Current-voltage relationship before and after prepulse on the same cells. **Note:** The prepulse dramatically decreased I_{to} (→) to the level of non significance between I_{to} L450F △ and I_{to} WT ▲). However, I_{Na} remained significantly decreased in presence of K_v4.3-L450F compared to WT. The trafficking-efficient K_v4.3-L450F leads to a significant decrease in I_{Na} for positive voltages to -35mV p<0.001.

Supplemental Figure 7: Raw traces of $Na_v1.5$+$K_v4.3$ in presence of β-subunits

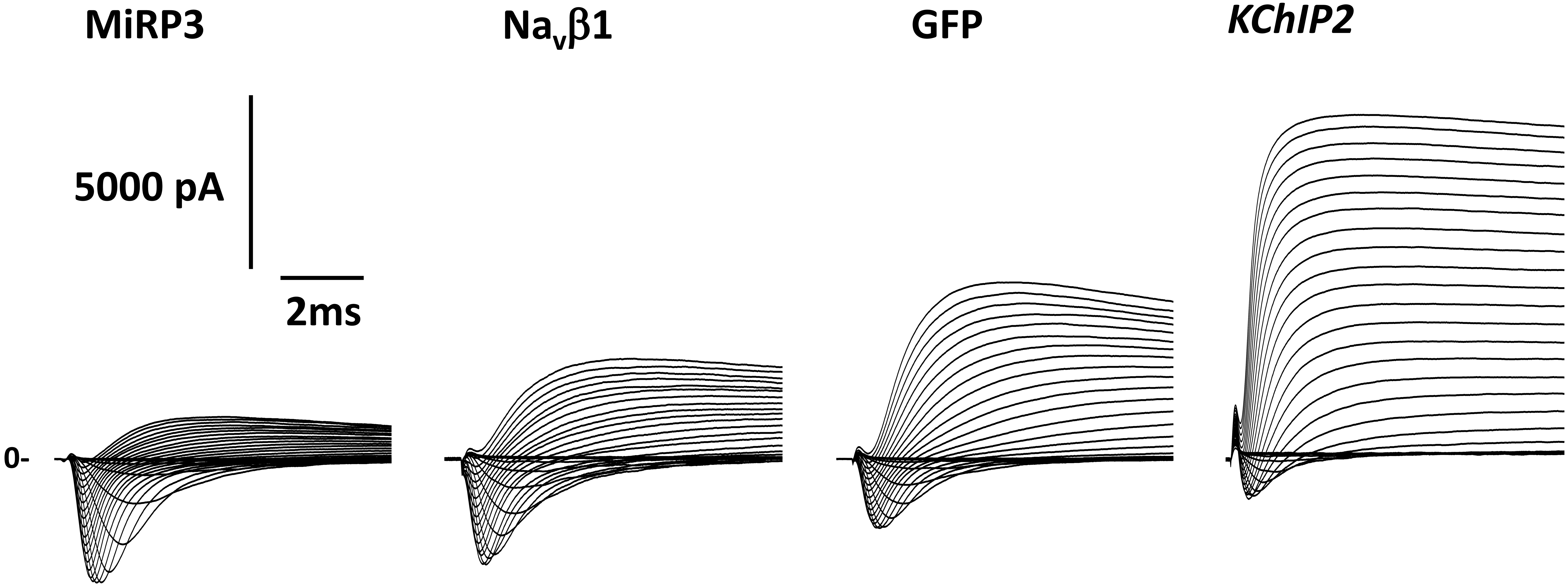

Representative raw traces of I_{to}/I_{Na} recorded in HEK293 cells expressing $K_v4.3$ and $Na_v1.5$ WT, in presence of β-subunits. **Note:** β-subunits increasing I_{Na} decrease I_{to} and reciprocally β-subunits that increase I_{to} decrease I_{Na}.

Supplemental Figure 8: Co-IP full blot

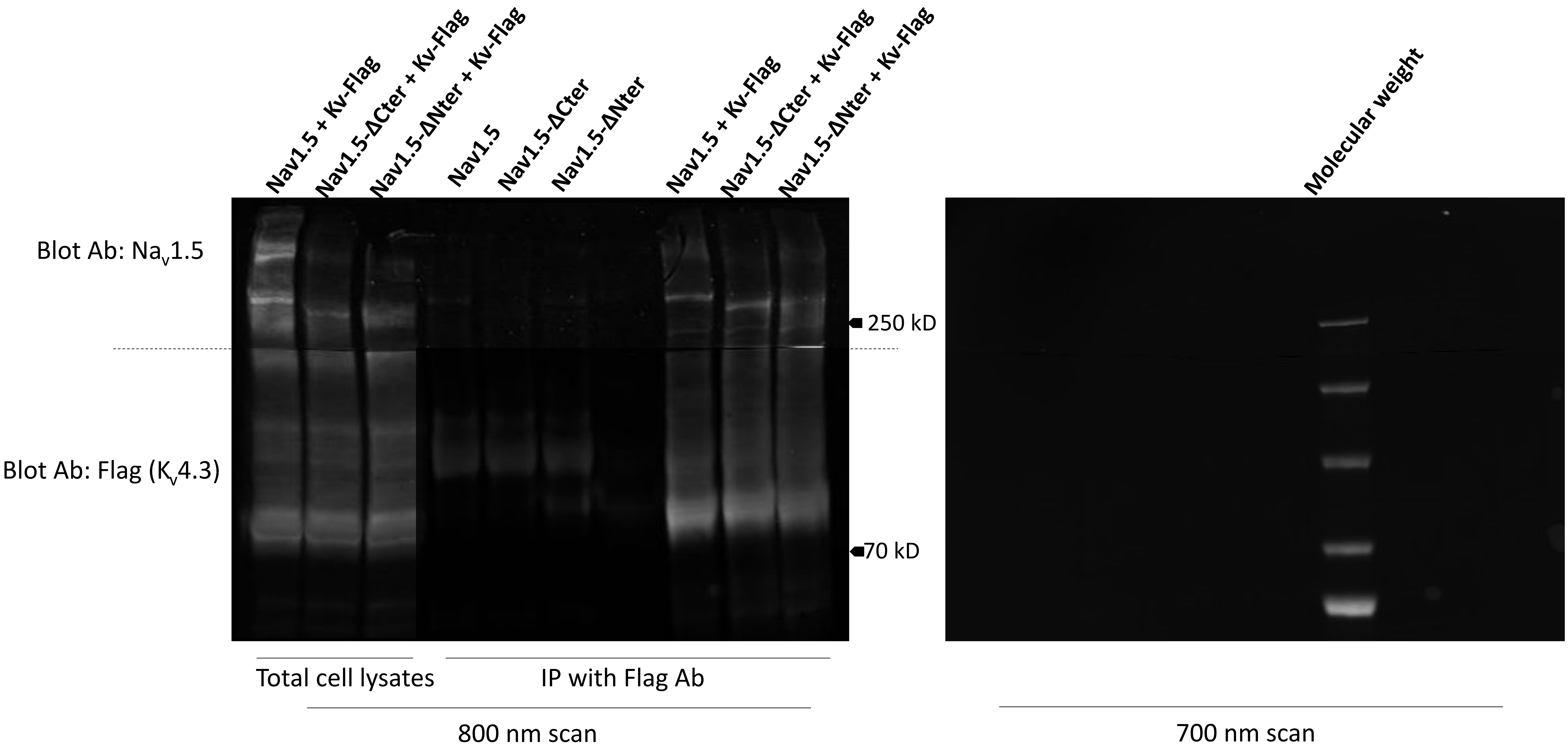

Figure: Co-immunoprecipitation of Nav1.5 constructs and Kv4.3

Co-Immunoprecipitation of Na_v1.5 constructs and K_v4.3 tagged with 3xFlag was performed in HEK293 cells. Na_v1.5, Na_v1.5-ΔNter, Na_v1.5-ΔCter or K_v4.3-3xFlag were transfected as indicated above the lanes. To assess interaction between Na_v1.5 constructs and K_v4.3, the total cell lysates were immunoprecipitated with anti-Flag antibody cross-linked to beads. The blots were hybridized with an anti-Na_v1.5 antibody (top gels: Blot Ab: Na_v1.5) or an anti-Flag antibody (bottom gels: Blot Ab: Flag). The left side corresponds to the total cell lysates of transfected cells before IP. The right side (IP with Flag Ab) corresponds to the elution fractions from beads. The results demonstrated an interaction between K_v4.3 and Na_v1.5 (n=7 different transfections), between K_v4.3 and Na_v1.5-ΔCter (n=4) and between K_v4.3 and Na_v1.5-ΔNter (n=4).

Supplemental Figure 9: Cell surface biotinylation full blots

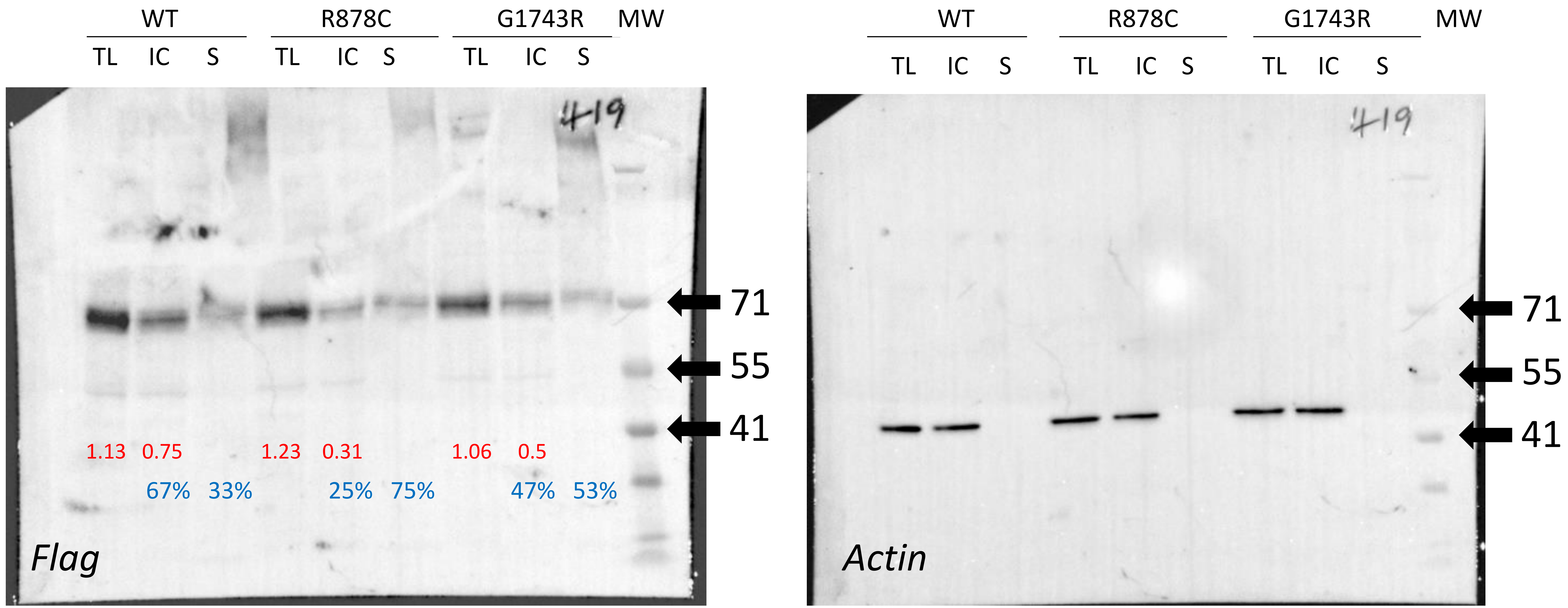

Cell surface biotinylation of Flag tagged-K_v4.3 proteins in HEK cells expressing Nav1.5-GFP WT vs Mutant. Proteins were biotinylated using EZ-Link Sulfo-NHS-S-S-Biotin as described in the Methods section. Proteins in the biotinylated (S) and non-biotinylated (IC) fractions along with total lysate were separated by Western blot, transferred to PVDF membranes then probed with anti-Flag (1:1000) followed by anti-actin (1:1000) antibodies. Luminescence (Clarity, BioRad) was detected using a ChemiDoc scanner (BioRad) and band density analyzed using Gen 5 software. TL = total lysate; IC = Intracellular fraction ; S = Surface fraction. Molecular Weight = MW. *Flag* signal intensity in the TL and IC fractions were quantitated using Adobe Photoshop and normalized to actin signal intensity (red numbers in *Flag* blot). IC intensity was calculated as a percentage of TL intensity and S determined by subtraction of the latter from 100% (blue numbers in *Flag* blot). S values were not directly quantitated from blots and are only shown to demonstrated that intracellular proteins were not biotinylated.
